# BRCA1–BARD1 Regulates Axon Regeneration in Concert with the Gqα–DAG Signaling Network

**DOI:** 10.1523/JNEUROSCI.1806-20.2021

**Published:** 2021-03-31

**Authors:** Yoshiki Sakai, Hiroshi Hanafusa, Tatsuhiro Shimizu, Strahil I. Pastuhov, Naoki Hisamoto, Kunihiro Matsumoto

**Affiliations:** Division of Biological Science, Graduate School of Science, Nagoya University, Nagoya 464-8602, Japan

**Keywords:** axon regeneration, BRCA1-BARD1, *Caenorhabditis elegans*, diacylglycerol kinase

## Abstract

The breast cancer susceptibility protein BRCA1 and its partner BRCA1-associated RING domain protein 1 (BARD1) form an E3-ubiquitin (Ub) ligase complex that acts as a tumor suppressor in mitotic cells. However, the roles of BRCA1–BARD1 in postmitotic cells, such as neurons, remain poorly defined. Here, we report that BRC-1 and BRD-1, the *Caenorhabditis elegans* orthologs of BRCA1 and BARD1, are required for adult-specific axon regeneration, which is positively regulated by the EGL-30 Gqα–diacylglycerol (DAG) signaling pathway. This pathway is downregulated by DAG kinase (DGK), which converts DAG to phosphatidic acid (PA). We demonstrate that inactivation of DGK-3 suppresses the *brc-1 brd-1* defect in axon regeneration, suggesting that BRC-1–BRD-1 inhibits DGK-3 function. Indeed, we show that BRC-1–BRD-1 poly-ubiquitylates DGK-3 in a manner dependent on its E3 ligase activity, causing DGK-3 degradation. Furthermore, we find that axon injury causes the translocation of BRC-1 from the nucleus to the cytoplasm, where DGK-3 is localized. These results suggest that the BRC-1–BRD-1 complex regulates axon regeneration in concert with the Gqα–DAG signaling network. Thus, this study describes a new role for breast cancer proteins in fully differentiated neurons and the molecular mechanism underlying the regulation of axon regeneration in response to nerve injury.

**SIGNIFICANCE STATEMENT** BRCA1–BRCA1-associated RING domain protein 1 (BARD1) is an E3-ubiquitin (Ub) ligase complex acting as a tumor suppressor in mitotic cells. The roles of BRCA1–BARD1 in postmitotic cells, such as neurons, remain poorly defined. We show here that *Caenorhabditis elegans* BRC-1/BRCA1 and BRD-1/BARD1 are required for adult-specific axon regeneration, a process that requires high diacylglycerol (DAG) levels in injured neurons. The DAG kinase (DGK)-3 inhibits axon regeneration by reducing DAG levels. We find that BRC-1–BRD-1 poly-ubiquitylates and degrades DGK-3, thereby keeping DAG levels elevated and promoting axon regeneration. Furthermore, we demonstrate that axon injury causes the translocation of BRC-1 from the nucleus to the cytoplasm, where DGK-3 is localized. Thus, this study describes a new role for BRCA1–BARD1 in fully-differentiated neurons.

## Introduction

Genetic susceptibility to breast cancer is caused largely by mutations in the *BRCA1* and *BRCA2* genes (Fackenthal and Olopade, 2007). These regulate a wide range of biological processes, including DNA damage repair by homologous recombination, gene silencing, cell cycle checkpoint, and centrosome duplication, all of which are relevant to the regulation of cell proliferation (Scully and Livingston, 2000; Moynahan and Jasin, 2010; Zhu et al., 2011; Li and Greenberg, 2012). BRCA1 exists primarily in a heterodimeric complex with the BRCA1-associated RING domain protein 1 (BARD1; Wu et al., 1996). It has been shown that this BRCA1–BARD1 complex possesses E3-ubiquitin (Ub) ligase activity, and this activity can be disrupted by cancer-derived mutations, underscoring the critical role of this enzymatic function in suppressing tumorigenesis (Baer and Ludwig, 2002). To date, intensive efforts have been devoted to understanding the tumor-suppressive functions of BRCA1–BARD1 and BRCA2 in mitotic cells. However, their roles in postmitotic cells, such as neurons, remain poorly understood at the molecular level.

Neurons are one type of postmitotic cell, specialized for transmitting information over long distances through axons. Although axons can be damaged by various internal and external stresses, neurons have a conserved system of regenerating axons postinjury, and failure of this system can cause sensory and motor paralysis. This axon's regenerative capacity is controlled by intrinsic neuronal signaling pathways (He and Jin, 2016). Upon axon injury, Ca^2+^ and cAMP levels rise in severed neurons, which drives various signaling pathways (Ghosh-Roy et al., 2010; Mar et al., 2014). For instance, cAMP elevation activates cAMP-dependent protein kinase A (PKA), which promotes axonal regeneration through phosphorylation of various downstream targets (Neumann et al., 2002; Bhatt et al., 2004; Gao et al., 2004). However, the intrinsic signaling pathways that regulate regeneration in the adult nervous system have yet to be fully elucidated.

The nematode *Caenorhabditis elegans* has recently emerged as an attractive model to dissect the mechanisms of axon regeneration in the mature nervous system (Yanik et al., 2004). Recent studies in *C. elegans* have identified many signaling molecules that promote or inhibit axon regeneration (Chen et al., 2011; Nix et al., 2014; Kim et al., 2018). We have previously demonstrated that the evolutionarily conserved JNK MAP kinase (MAPK) pathway, consisting of MLK-1 MAPKKK–MEK-1 MAPKK–KGB-1 JNK, drives the initiation of axon regeneration (Nix et al., 2011). Two different protein kinases act as MAP4Ks for MLK-1 in a manner specific for different life stages. The Ste20-related kinase MAX-2 phosphorylates and activates MLK-1 mainly at the L4 stage to promote axon regeneration (Pastuhov et al., 2016). On the other hand, the protein kinase C (PKC) ortholog TPA-1 can activate MLK-1 at the young adult stage, but not at the L4 stage (Pastuhov et al., 2012). The Gqα protein EGL-30 acts as a component upstream of TPA-1. EGL-30 activates the phospholipase Cβ (PLCβ) EGL-8, which in turn generates diacylglycerol (DAG), an activator of TPA-1, from phosphatidylinositol bisphosphate (Lackner et al., 1999). DAG kinases (DGKs) antagonize the EGL-30 pathway by converting DAG to phosphatidic acid (PA; Miller et al., 1999).

**Figure 1.**
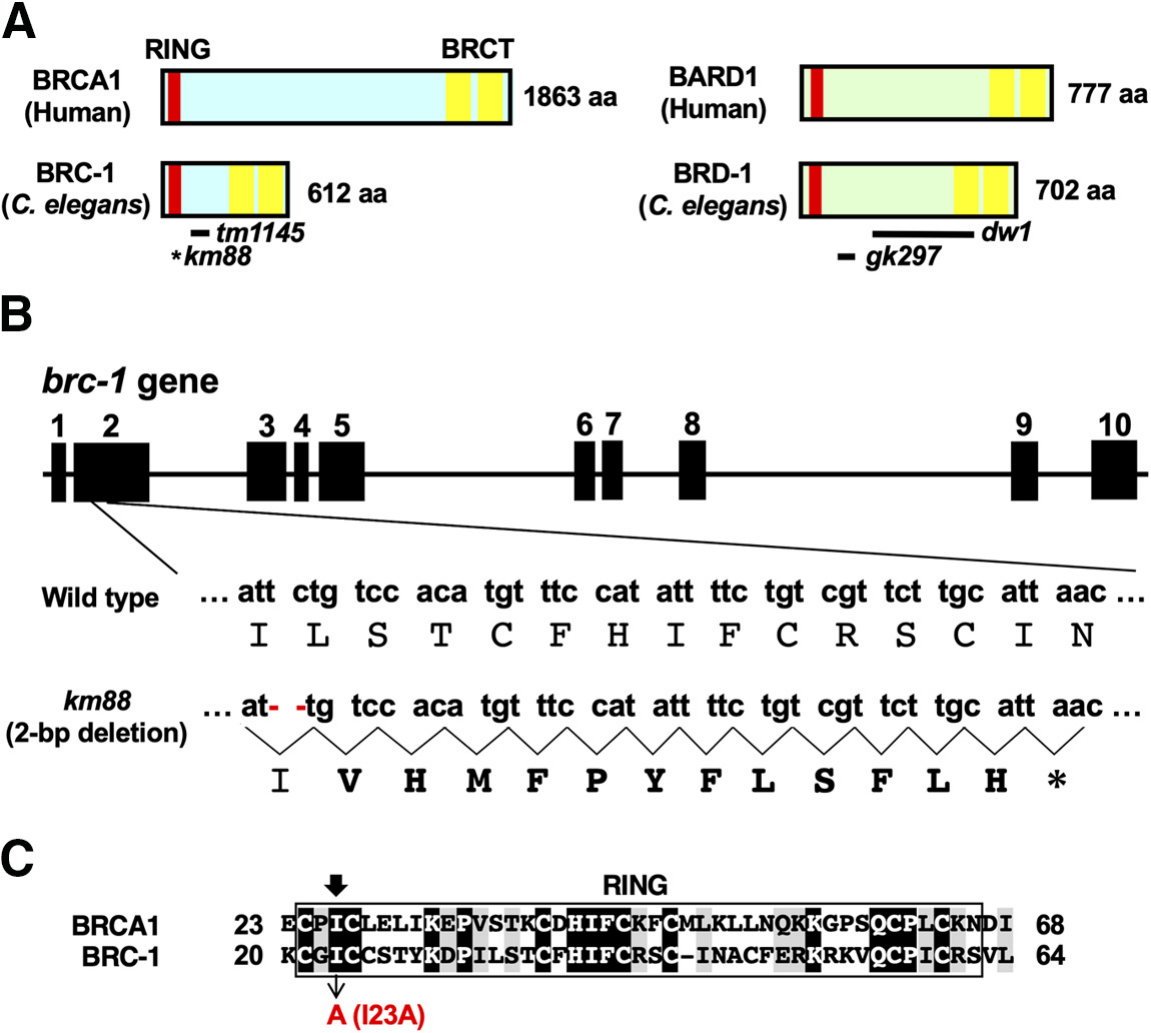
*C. elegans* BRC-1 and BRD-1. ***A***, Structures of BRC-1 and BRD-1. Schematic diagrams of BRC-1, BRD-1, and their mammalian counterparts, BRCA1 and BARD1, are shown. RING finger domain is shown in red and BRCT domains in yellow. The bold lines underneath denote the extent of the deleted regions in the *tm1145*, *dw1*, and *gk297* mutants. An asterisk indicates a premature stop codon caused by the *km88* mutation. ***B***, Isolation of *brc-1* mutants. Genomic structure of the *brc-1* gene is shown. Exons are indicated by boxes, and introns and untranslated regions are indicated by bars. Small and capital letters indicate nucleotides and the corresponding amino acids, respectively. The *brc-1(km88)* mutation is a 2-bp deletion, causing a frameshift (bold amino acids) and premature stop codon (*) in exon 2. ***C***, Ring finger domain. Sequence alignment in the RING finger domain between BRCA1 and BRC-1 is shown. Identical and similar residues are highlighted with black and gray shading, respectively. The black arrow indicates the conserved isoleucine residue required for E3-Ub ligase activity.

We have recently found that BRC-2, the *C. elegans* ortholog of BRCA2, acts as a regulator of axon regeneration (Shimizu et al., 2018). *C. elegans* also has two genes, *brc-1* and *brd-1*, which encode orthologs of mammalian BRCA1 and BARD1, respectively (Fig. 1*A*; Boulton et al., 2004). BRC-1 and BRD-1 share extensive sequence and domain conservation with their mammalian counterparts, including RING and BRCT domains. Similar to mammalian BRCA1–BARD1, BRC-1 heterodimerizes with BRD-1 to form a complex having E3-Ub ligase activity (Polanowska et al., 2006). BRC-1–BRD-1 is involved in DNA repair at sites damaged by ionizing radiation. Our finding that BRC-2 is implicated in axon regeneration prompted us to explore the possibility that BRC-1 and BRD-1 also participate in this process.

In this study, we investigated the roles of BRC-1 and BRD-1 in axon regeneration. We found that the BRC-1–BRD-1 complex is required for axon regeneration after injury, specifically in the adult stage. We demonstrate that BRC-1–BRD-1 poly-ubiquitylates DGK-3, resulting in its degradation. Thus, BRC-1–BRD-1 enhances the EGL-30 signaling pathway by downregulating DGK-3 to promote axon regeneration. Furthermore, we show that PKA phosphorylates BRC-1, which causes the translocation of BRC-1 from the nucleus to the cytoplasm, where DGK-3 is localized. These results suggest that the BRC-1–BRD-1 complex regulates axon regeneration in concert with the Gqα–DAG signaling network. Thus, this study uncovers an unexpected role of BRC-1–BRD-1 in postmitotic neurons and suggests a molecular mechanism by which BRC-1–BRD-1 regulates axon regeneration in response to nerve injury.

## Materials and Methods

### 

#### C. elegans strains

The *C. elegans* strains used in this study are listed in Table 1. All strains were maintained on nematode growth medium plates and fed with bacteria of the OP50 strain by the standard method (Brenner, 1974).

**Table 1.** Strains used in this study

Strain	Genotype
KU501	*juIs76 II*
KU88	*juIs76 II; brc-1(km88) III*
KU1440	*juIs76 II; brc-1(km88) III; kmEx1440 [Punc-25::brc-1]*
KU1441	*juIs76 II; brc-1(km88) III; kmEx1441 [Punc-25::brc-1(I23A)]*
KU1442	*juIs76 II; brd-1(gk297) III*
KU1443	*juIs76 II; brc-1(tm1145) brd-1(dw1) III*
KU1444	*juIs76 II; kmEx1444 [Punc-25::brc-1* + *Punc-25::brd-1 (line 1)]*
KU1445	*juIs76 II; kmEx1445 [Punc-25::brc-1* + *Punc-25::brd-1 (line 2)]*
KU456	*egl-30(ad805) I; juIs76 II*
KU457	*egl-30(tg26) I; juIs76 II*
KU461	*juIs76 II; tpa-1(k501) IV*
KU1446	*egl-30(ad805) I; juIs76 II; brc-1(tm1145) brd-1(dw1) III*
KU1447	*egl-30(tg26) I; juIs76 II; brc-1(tm1145) brd-1(dw1) III*
KU1448	*goa-1(n1134) I; juIs76 II; brc-1(tm1145) brd-1(dw1) III*
KU1449	*eat-16(nj8) I; juIs76 II; brc-1(tm1145) brd-1(dw1) III*
KU1450	*juIs76 II; brc-1(tm1145) brd-1(dw1) III; dgk-1(ok1462) X*
KU89	*juIs76 II; dgk-3(km89) III*
KU1451	*juIs76 II; brc-1(tm1145) brd-1(dw1) dgk-3(km89) III*
KU1452	*juIs76 II; brc-1(tm1145) brd-1(dw1) dgk-3(km89) III; dgk-1(ok1462) X*
KU1453	*juIs76 II; brc-1(tm1145) brd-1(dw1) dgk-3(km90) III; dgk-1(ok1462) X*
KU1454	*juIs76 II; brc-1(tm1145) brd-1(dw1) dgk-3(km89) III; tpa-1(k501) IV*
KU1455	*juIs76 II; brc-1(S266A) III*
KU1456	*wpIs36 I; kmEx1456 [Punc-25::dgk-3::gfpnovo_2_]*
KU1457	*wpIs36 I; brc-1(tm1145) brd-1(dw1) III; kmEx1456 [Punc-25::dgk-3::gfpnovo_2_]*
KU1458	*wpIs36 I; kmEx1458 [Punc-25::gfpnovo_2_::brc-1]*
KU1459	*wpIs36 I; kmEx1459 [Punc-25::gfpnovo_2_::brc-1(S266A)]*
KU1343	*muIs32 II*
KU1460	*muIs32 II; brc-1(tm1145) brd-1(dw1) III*

#### Plasmids

*Punc-25::brc-1* and *Punc-25::brd-1* were respectively generated by inserting *brc-1* cDNA (isoform a) and *brd-1* cDNA isolated from cDNA library into a pSC325 vector, respectively. *Punc-25::gfpnovo2::brc-1* was generated by inserting the GFPnovo2 coding sequence isolated from the pSM-GFPnovo2 plasmid into *Punc-25::brc-1*. *Punc-25::brc-1(I23A)* and *Punc-25::gfpnovo2::brc-1(S266A)* were generated by oligonucleotide-directed PCR using *Punc-25::brc-1* and *Punc-25::gfpnovo2::brc-1* as templates, respectively, and the mutations were verified by DNA sequencing. *Punc-25::dgk-3::gfpnovo2* was generated by inserting the *dgk-3* cDNA and the GFPnovo2 coding sequence, which were isolated from a cDNA library and the pSM-GFPnovo2 plasmid, respectively, into the pSC325 vector. The T7-DGK-3, GFP-BRC-1, and BRD-1-RFP plasmids were generated by inserting the *dgk-3*, *brc-1* and *brd-1* cDNAs into the pCMV-T7, pEGFP-C1, and pTagRFP-N vectors, respectively. GFP-BRC-1(I23A) was generated by oligonucleotide-directed PCR using GFP-BRC-1 as a template, and the mutation was verified by DNA sequencing. The *Pmyo-2::dsred-monomer*, and HA-Ub plasmids were described previously (Hanafusa et al., 2011; Li et al., 2012).

#### Generation of the brc-1 and dgk-3 mutations using CRISPR–Cas9

The *brc-1* mutations (*km88* deletion and S266A point mutation) and the *dgk-3* mutations (*km89* insertion and *km90* deletion) were obtained using the CRISPR–Cas9 system as described previously (Dokshin et al., 2018). The CRISPR RNAs [5′-UGGAAACAUGUGGACAGAAU-3′ for *brc-1(km88)*, 5′-UUGCGAGUUCUCAAGAUCUU-3′ for *brc-1(S266A)*, and 5′-UAUCACCGGAGCAAUUCUCG-3′ for *dgk-3(km89, km90)*] and the single-stranded donor template DNA [5′-ATCAGAGAAACCAGCGAATCGAAGAGTAgccTTTGCGAGTTCTCAAGATCTTGAAAACA\TAAAAATTATG-3′ for *brc-1(S266A)*] were synthesized (Integrated DNA Technologies; IDT), co-injected with the trans-activating CRISPR RNA (IDT), Streptococcus pyogenes Cas9 3NLS (IDT) protein, and the pRF4(rol-6d) plasmid into the KU501 [for *brc-1(km88)* and *brc-1(S266A)*] and KU1448 [for *dgk-3(km89*, *km90)*] strains. Each of the F1 animals carrying the transgene was transferred onto a new dish and used for single-worm PCR, followed by DNA sequencing to detect the mutations. The *brc-1(km88)* mutation is a 2-bp deletion in the *brc-1* gene, causing a frameshift and premature stop codon in exon 2. The *dgk-3(km89)* mutation is a 20-bp insertion that contains an in-frame stop codon, thus terminating translation in the middle of exon 1. The *dgk-3(km90)* mutation is a 5-bp deletion, causing a frameshift and premature stop codon in exon 1.

#### Transgenic animals

Transgenic animals were obtained using the standard *C. elegans* microinjection method (Mello et al., 1991). *Pmyo-2::dsred-monomer*, *Punc-25::brc-1*, *Punc-25::brc-1(I23A)*, *Punc-25::brd-1, Punc-25::dgk-3::gfpnovo2*, *Punc-25::gfpnovo2::brc-1*, and *Punc-25::gfpnovo2::brc-1(S266A)* plasmids were used in *kmEx1440* [*Punc-25::brc-1* (5 ng/µl) + *Pmyo-2::dsred-monomer* (5 ng/µl)], *kmEx1441* [*Punc-25::brc-1(I23A)* (5 ng/µl) + *Pmyo-2::dsred-monomer* (5 ng/µl)], *kmEx1444*/*kmEx1445* [*Punc-25::brc-1* (25 ng/µl) + *Punc-25::brd-1* (25 ng/µl) + *Pmyo-2::dsred-monomer* (5 ng/µl)], *kmEx1456* [*Punc-25::dgk-3::gfpnovo2* (5 ng/µl) + *Pmyo-2::dsred-monomer* (5 ng/µl)], *kmEx1458* [*Punc-25::gfpnovo2::brc-1* (10 ng/µl)+ *Pmyo-2::dsred-monomer* (5 ng/µl)], *kmEx1459* [*Punc-25::gfpnovo2::brc-1(S266A)* (10 ng/µl) + *Pmyo-2::dsred-monomer* (5 ng/µl)], respectively. The *juIs76*, *wpIs36*, and *muIs32* integrated arrays were described previously (Huang et al., 2002; Ch'ng et al., 2003; Firnhaber and Hammarlund, 2013).

#### Microscopy

Standard fluorescent images of transgenic animals were observed under an 100× objective of a Nikon ECLIPSE E800 fluorescent microscope and photographed with a Zyla CCD camera. Confocal fluorescent images were taken on a Zeiss LSM-800 confocal laser-scanning microscope with a 63× objective.

#### Axotomy

Axotomy and microscopy were performed as described previously (Li et al., 2012). Animals were subjected to axotomy at the young adult or L4 stage. The young adult stage was defined as a state in which the vulva is well developed and no eggs have formed yet. Imaged commissures that had growth cones or small branches present on the proximal fragment were counted as “regenerated.” Proximal fragments that showed no change after 24 h were counted as “no regeneration.” A minimum of 20 individuals with one to three axotomized commissures were observed for most experiments.

#### Measurements of regenerating axons

The length of regenerating axons for either D-type motor neurons or touch sensory posterior lateral microtubule (PLM) neurons was measured using the segmented line tool of ImageJ. Measurements were made from the site of injury to the tip of the longest branch of the regenerating axon. Axons that did not regenerate were excluded. Data were plotted using R (ver. 4.0.1) and R studio (ver. 1.3.959).

#### Immunoprecipitation

For immunoprecipitation, transfected COS-7 cells that were incubated with or without MG132 (Sigma; 10 μm) for 8 h were lysed in RIPA buffer [50 mm Tris–HCl, pH 7.4, 0.15 m NaCl, 0.25% deoxycholic acid, 1% NP-40, 1 mm EDTA, 1 mm dithiothreitol, 1 mm phenylmethylsulfonyl fluoride, phosphatase inhibitor cocktail 2 and 3 (Sigma), and protease inhibitor cocktail (Sigma)], followed by centrifugation at 15,000 × *g* for 12 min. A total of 10 µl (bed volume) of Dynabeads Protein G (Invitrogen) with anti-T7 antibody (PM022; MBL) was added to supernatant and the sample was rotated for 2 h at 4°C. The beads were then washed three times with ice-cold PBS and subjected to immunoblotting.

#### Immunoblotting

After cell extracts were subjected to SDS-PAGE, proteins were transferred to a polyvinylidene difluoride membrane (Hybond-P; GE Healthcare). The membranes were immunoblotted with anti-HA antibody (mouse 16B12; BioLegend), anti-T7 antibody (mouse T7-Tag; Merck; or rabbit PM022; MBL), anti-GFP antibody (mouse JL-8; Clontech), or anti-RFP antibody (rabbit AB233; Evrogen), and bound antibodies were visualized with horseradish peroxidase (HRP)-conjugated antibodies against rabbit or mouse IgG using an HRP chemiluminescent substrate reagent kit (Novex ECL; Invitrogen).

#### In vitro kinase assays

GFP-BRC-1 proteins were immunopurified from transfected COS-7 cells using anti-GFP antibody (mouse M048-3; MBL). Kinase reactions were performed in a final volume of 20 µl in buffer consisting of 25 mm MOPS (pH 7.2), 12.5 mm glycerol phosphate, 25 mm MgCl_2_, 2 mm EDTA, 0.25 mm DTT, 200 μm ATP, and 0.4 µg of recombinant PKA (Carna Biosciences). Samples were incubated for 20 min at 30°C and the reactions were terminated by the addition of Laemmli sample buffer and boiling. Phosphorylation of BRC-1 was detected by immunoblotting with rabbit anti-phospho-PKA substrate antibody (100G7E; Cell Signaling).

#### Forskolin treatment

Treatment of animals with forskolin was performed as described previously (Ghosh-Roy et al., 2010). Forskolin (ab120058; Abcam) dissolved in DMSO was diluted in M9 media (500 mm). L4 stage worms were incubated in the forskolin solution (containing heat-killed OP50) for 12 h followed by fluorescent microscopic observation.

#### Quantification of DGK-3 poly-ubiquitylation

To compare differences in DGK-3 poly-ubiquitylation, band intensity minus background of HA (Ub) and T7 (DGK-3) was quantified in lanes 4 and 5 using the FUSION system (VILBER). The HA (Ub) value was divided by the corresponding T7 (DGK-3) value to determine a normalized HA (Ub) value for lanes 4 and 5. To compare DGK-3 poly-ubiquitylation levels between lanes 4 and 5, normalized HA (Ub) values in lane 5 were divided by the values in lane 4, and the derived ratios were plotted on a bar graph.

#### Quantitative measures of fluorescence intensity for DGK-3 degradation

Animals expressing mCherry and DGK-3::GFP in D-type motor neurons were imaged immediately after axotomy (0 h) and 8 h after axotomy of selected motor neuron axons. A LSM800 confocal microscope (Zeiss) was used to obtain a z-stack of fluorescent images for mCherry and DGK-3::GFP. Mean intensity of DGK-3::GFP and mCherry in cytoplasm of neurons with severed axons was measured by drawing a circular region of interest in the center of the cell and using the measure function of ImageJ. Background intensity was determined near analyzed cells. Relative DGK-3::GFP intensity (RI_DGK-3_) was obtained by dividing the background-subtracted value of GFP by the corresponding background-corrected value of mCherry, followed by dividing the value 8 h after axotomy by the corresponding value 0 h after axotomy. The RI_DGK-3_ values for wild-type and *brc-1 brd-1* mutants were plotted and checked for significant differences by Wilcoxon rank-sum test using R (ver. 4.0.1) and R studio (ver. 1.3.959).

#### Quantitative measures of fluorescence intensity for BRC-1 localization

Animals expressing mCherry and GFP-BRC-1 in D-type motor neurons with or without forskolin treatment were imaged using a Nikon ECLIPSE E800 fluorescent microscope and Zyla CCD camera. Mean intensities of GFP-BRC-1 and mCherry were measured in the cytoplasm and nucleus of D-type neurons, respectively. Background intensity was determined by measuring the mean GFP (or mCherry) intensity of adjacent regions of the same size. Normalized cytoplasmic and nuclear GFP-BRC-1 values were calculated by dividing background-subtracted cytoplasmic or nuclear GFP-BRC-1 by the corresponding background-corrected mCherry intensity. To compare cytoplasmic and nuclear GFP-BRC-1, a cytoplasmic-to-nuclear ratio was calculated and plotted using R (ver. 4.0.1) and R studio (ver. 1.3.959).

#### Experimental design and statistical analyses

All experiments were not randomized and the investigators were not blinded to the group allocation during experiments and outcome assessment. No statistical methods were used to predetermine sample size. Data visualization was performed using Microsoft Excel 2016, R (ver. 4.0.1), and R studio (ver. 1.3.959). Statistical analysis was conducted as described previously (Pastuhov et al., 2012). Briefly, 95% confidence intervals were calculated using the modified Wald method, and the two-tailed *p* values were calculated using Fisher's exact test on GraphPad QuickCalcs (http://www.graphpad.com/quickcalcs/contingency1/). The Wilcoxon rank-sum test (two-tailed) was performed using R (ver. 4.0.1), R studio (ver. 1.3.959), and the R exactRankTests package.

## Results

### BRC-1 and BRD-1 are required for axon regeneration

**Figure 2.**
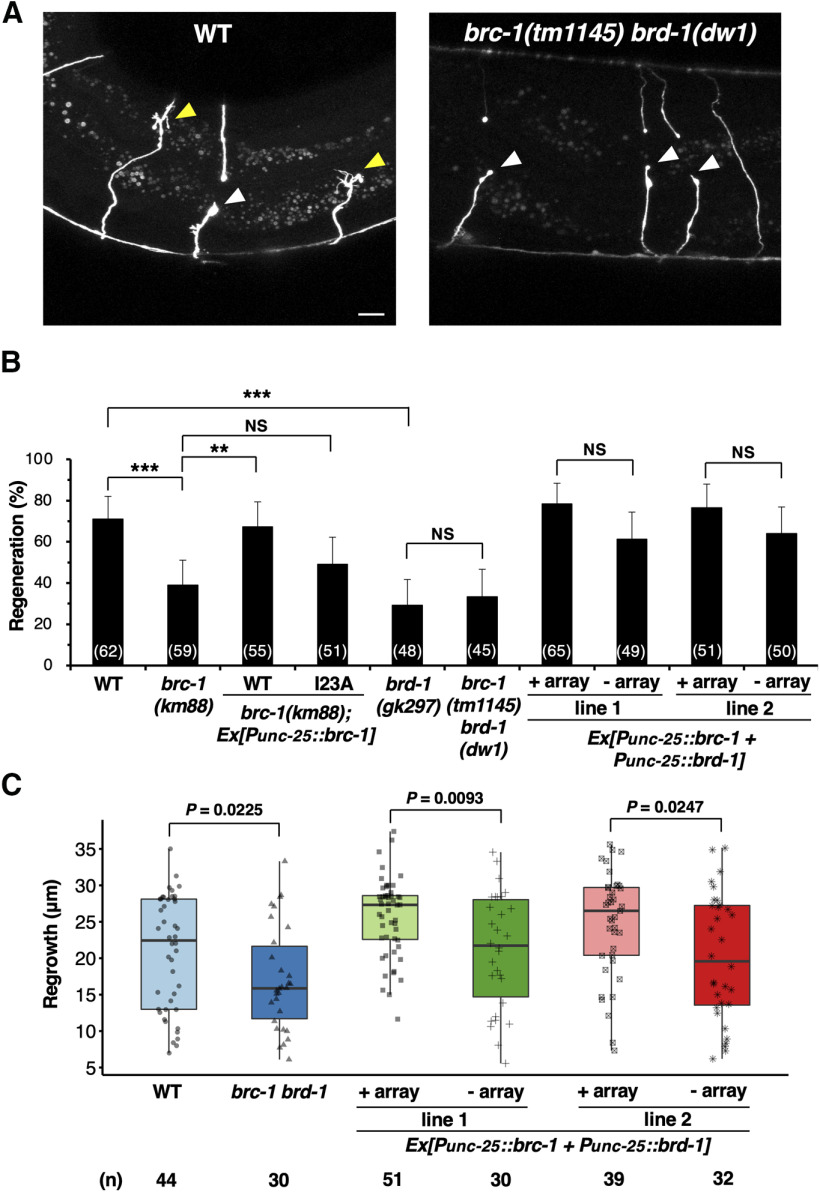
BRC-1 and BRD-1 are required for axon regeneration of D-type motor neurons. ***A***, Representative D-type motor neurons in wild-type and *brc-1(tm1145) brd-1(dw1)* mutant animals 24 h after laser surgery. In wild-type animals, severed axons exhibited regenerated growth cones (yellow arrowheads). In *brc-1(tm1145) brd-1(dw1)* mutants, the proximal ends of axons failed to regenerate (white arrowheads). Scale bar: 10 µm. ***B***, Percentages of axons that initiated regeneration 24 h after laser surgery at the young adult stage. The number of axons examined is shown. Error bars indicate 95% confidence intervals; **p < 0.01, ***p < 0.001, as determined by Fisher's exact test. NS, not significant. ***C***, Length of regenerating axons 24 h after laser surgery. Data are presented as a box-plot representing median (thick line within the box) and interquartile range (edge of box) with individual data points. The number (*n*) of axons examined is shown. Statistical significance was determined by Wilcoxon rank-sum test.

To assess whether the BRCA1 ortholog BRC-1 is involved in axon regeneration, we used the CRISPR–Cas9 system to generate the null mutant *brc-1(km88)*, which harbors a 2-bp deletion generating a premature stop codon in the second exon of the *brc-1* gene (Fig. 1*A*,*B*). We first assayed regrowth following laser axotomy in GABA-releasing D-type motor neurons (Fig. 2*A*). In young adult wild-type animals, ∼70% of the axons initiated regeneration within 24 h after axon injury (Fig. 2*A*,*B*; Table 2). However, in *brc-1(km88)* mutants the frequency of axon regeneration was significantly reduced (Fig. 2*B*; Table 2). This indicates that BRC-1 is required for efficient axon regeneration following laser axotomy. To test whether BRC-1 can act in a cell-autonomous manner, we expressed the *brc-1* cDNA from the *unc-25* promoter in *brc-1* mutants. We found that the axon regeneration defect of *brc-1(km88)* mutants was rescued by expression of *brc-1* in D-type motor neurons (Fig. 2*B*; Table 2). These results demonstrate that BRC-1 functions cell autonomously in injured neurons.

**Table 2.** Raw data for genotypes tested by axotomy

Strain	Genotype (*juIs76* background)	Age	Number of axons	Number of regenerations (% of total)	*p* value	Compared with
KU501*^a^*^1^	Wild type	YA	62	44 (71%)	-	-
KU88	*brc-1(km88)*	YA	59	23 (39%)	0.0005	KU501*^a^*^1^
KU1440	*brc-1(km88); kmEx1440 [Punc-25::brc-1]*	YA	55	37 (67%)	0.0029	KU88
KU1441	*brc-1(km88); kmEx1441 [Punc-25::brc-1(I23A)]*	YA	51	25 (49%)	0.3374	KU88
KU1442	*brd-1(gk297)*	YA	48	14 (29%)	<0.0001	KU501*^a^*^1^
KU1443*^a^*	*brc-1(tm1145) brd-1(dw1)*	YA	45	15 (33%)	0.8231	KU1442
KU501*^a^*^2^	Wild type(–kmEx1444)	YA	49	30 (61%)	-	-
KU1444	*kmEx1444 [Punc-25::brc-1* + *Punc-25::brd-1 (line 1)]*	YA	65	51 (78%)	0.0604	KU501*^a^*^2^
KU501*^a^*^3^	Wild type(–kmEx1445)	YA	50	32 (64%)	-	-
KU1445	*kmEx1445 [Punc-25::brc-1* + *Punc-25::brd-1 (line 2)]*	YA	51	39 (76%)	0.1961	KU501*^a^*^3^
KU501*^b^*	Wild type	L4	57	40 (70%)	-	-
		YA	74	50 (68%)	-	-
KU1443*^b^*	*brc-1(tm1145) brd-1(dw1)*	L4	71	51 (72%)	0.8471	KU501*^b^* (L4)
		YA	53	19 (36%)	0.0006	KU501*^b^* (YA)
KU456	*egl-30(ad805)*	L4	41	31 (76%)	0.2688	KU501*^b^* (L4)
		YA	50	20 (40%)	0.0031	KU501*^b^* (YA)
KU457	*egl-30(tg26)*	YA	30	21 (70%)	1.0000	KU501*^b^* (YA)
KU461	*tpa-1(k501)*	L4	49	34 (69%)	1.0000	KU501*^b^* (L4)
		YA	47	17 (36%)	0.0013	KU501*^b^* (YA)
KU1446	*egl-30(ad805); brc-1(tm1145) brd-1(dw1)*	YA	50	17 (34%)	1.0000	KU1443*^b^*(YA)
					0.6790	KU456(YA)
KU1447	*egl-30(tg26); brc-1(tm1145) brd-1(dw1)*	YA	94	61(65%)	0.0010	KU1443*^b^*(YA)
KU501*^c^*	Wild type	YA	60	37 (62%)	-	-
KU1443*^c^*	*brc-1(tm1145) brd-1(dw1)*	YA	48	18 (38%)	0.0197	KU501*^c^*
KU1448	*goa-1(n1134); brc-1(tm1145) brd-1(dw1)*	YA	58	20 (34%)	0.8395	KU1443*^c^*
KU1449	*eat-16(nj8); brc-1(tm1145) brd-1(dw1)*	YA	51	13 (25%)	0.2783	KU1443*^c^*
KU1450	*brc-1(tm1145) brd-1(dw1); dgk-1(ok1462)*	YA	56	21 (38%)	1.0000	KU1443*^c^*
KU1452	*brc-1(tm1145) brd-1(dw1) dgk-3(km89); dgk-1(ok1462)*	YA	60	38 (63%)	0.0115	KU1443*^c^*
KU1453	*brc-1(tm1145) brd-1(dw1) dgk-3(km90); dgk-1(ok1462)*	YA	53	35 (66%)	0.0053	KU1443*^c^*
KU1451	*brc-1(tm1145) brd-1(dw1) dgk-3(km89)*	YA	45	30 (67%)	0.0069	KU1443*^c^*
KU1454	*brc-1(tm1145) brd-1(dw1) dgk-3(km89); tpa-1(k501) IV*	YA	59	25 (42%)	0.6931	KU1443*^c^*
KU89	*dgk-3(km89)*	YA	64	51 (80%)	0.0310	KU501*^c^*
KU501*^d^*	Wild type	YA	55	38 (69%)	-	-
KU1455	*brc-1(S266A)*	YA	61	23 (38%)	0.0008	KU501*^d^*

We next asked whether the BARD1 ortholog BRD-1 also participates in axon regeneration. We found that the *brd-1(gk297)* deletion (Fig. 1*A*) markedly reduced axon regrowth following laser injury (Fig. 2*B*; Table 2). Furthermore, we observed that the regeneration defect observed in *brc-1(tm1145) brd-1(dw1)* double mutants (Fig. 1*A*) was no greater than that seen in the single *brd-1(gk297)* mutant (Fig. 2*B*; Table 2), suggesting that BRC-1 and BRD-1 act in the same pathway. This suggests that BRC-1 and BRD-1 function as a complex to regulate axon regeneration.

We investigated the effects of *brc-1* and *brd-1* on growth cone behavior, and found that the length of regenerated axons in *brc-1(tm1145) brd-1(dw1)* mutants was shorter than observed in wild-type animals (Fig. 2*C*). In contrast, when both *brc-1* and *brd-1* were overexpressed using the *unc-25* promoter, regenerated axons were longer than those in wild-type animals (Fig. 2*C*). In fact, 28% (25/90) of regenerated axons reached the dorsal nerve cord of animals overexpressing *brc-1* and *brd-1* compared with 11% (7/62) in wild-type adult animals. Overexpression of *brc-1*/*brd-1* appeared to increase the frequency of axon regeneration, but the difference was not statistically significant (Fig. 2*B*; Table 2). Thus, BRC-1–BRD-1 is required to initiate axon regeneration and control growth cone behavior.

**Figure 3.**
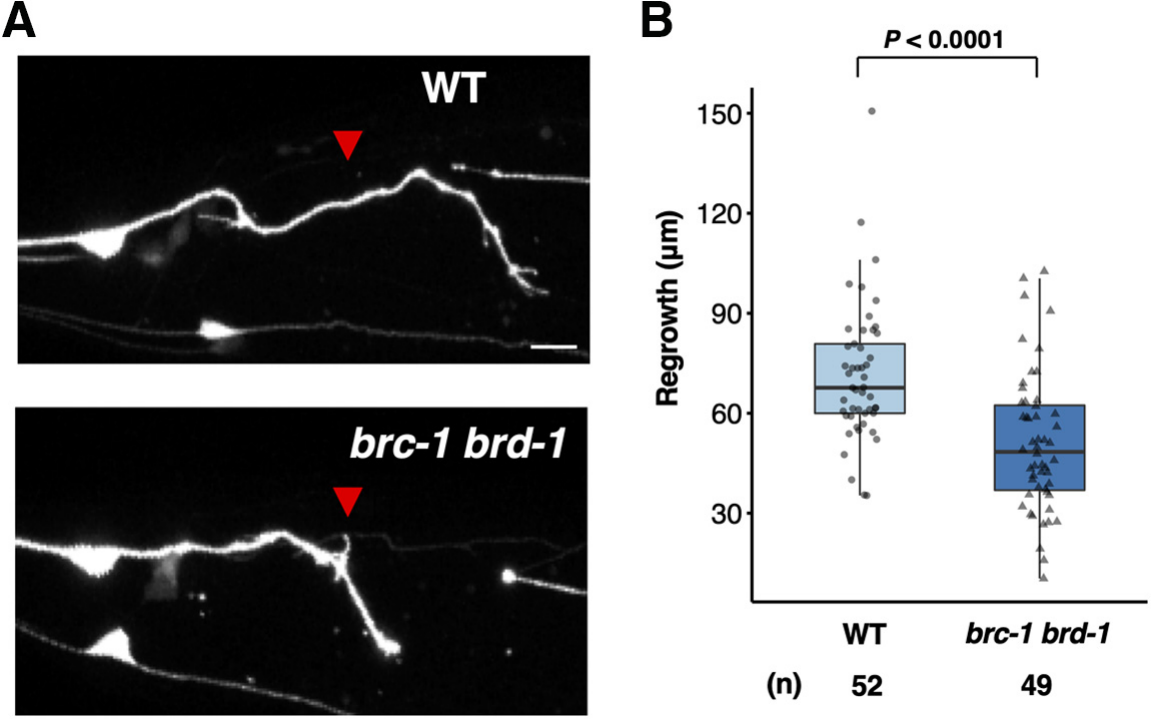
BRC-1 and BRD-1 are required for axon regeneration of PLM sensory neurons. ***A***, Representative PLM sensory neurons in wild-type and *brc-1(tm1145) brd-1(dw1)* mutant animals 24 h after laser surgery. Red arrowheads indicate cut sites. Scale bar: 10 µm. ***B***, Length of PLM regrowth 24 h after laser surgery. Data are presented as a box-plot representing median (thick line within the box) and interquartile range (edge of box) with individual data points. The number (*n*) of axons examined is shown. Statistical significance was determined by Wilcoxon rank-sum test.

Next, to determine whether the effect of BRC-1–BRD-1 complex on axon regeneration is specific to D-type motor neurons, we examined the effect of *brc-1* and *brd-1* on axon regeneration in glutaminergic touch sensory PLM neurons (Fig. 3*A*). Chen et al. (2011) previously performed a systematic mutant screen looking for defects in axon regeneration, and identified *brd-1* as a positive regulator of axon regeneration in PLM neurons. Consistent with their finding, we found that *brc-1(tm1145) brd-1(dw1)* mutants were defective in axon regeneration in PLM neurons (Fig. 3*A*,*B*). These results suggest that BRC-1–BRD-1 is generally required by neurons for axon regeneration.

BRCA1 contains a RING finger domain that functions as an E3-Ub ligase *in vitro*. This activity is greatly increased when complexed with BARD1, which also harbors a RING domain (Fig. 1*A*; Baer and Ludwig, 2002). The Ile-26 residue in the BRCA1 RING domain is essential for its interaction with the E2-Ub conjugating enzyme but not for its interaction with BARD1, suggesting that BRCA1 is the critical subunit required for E3-Ub ligase activity. Accordingly, the I26A mutant, in which Ile-26 was replaced with alanine, is defective in E3-Ub ligase activity (Brzovic et al., 2003). Similar to mammalian BRCA1, BRC-1 possesses a RING domain with a conserved site, Ile-23, corresponding to the mammalian Ile-26 (Fig. 1*C*). To determine the importance of BRC-1 E3-Ub ligase activity in axon regeneration, we generated a mutant form of BRC-1 [BRC-1(I23A)] with Ile-23 mutated to alanine. We found that the I23A point mutation could not rescue the *brc-1(km88)* phenotype (Fig. 2*B*; Table 2). Taken together, these results suggest that the BRC-1–BRD-1 complex is required for axon regeneration in a manner dependent on its E3-Ub ligase activity.

### BRC-1–BRD-1 functions in the EGL-30 Gqα signaling pathway to regulate axon regeneration

**Figure 4.**
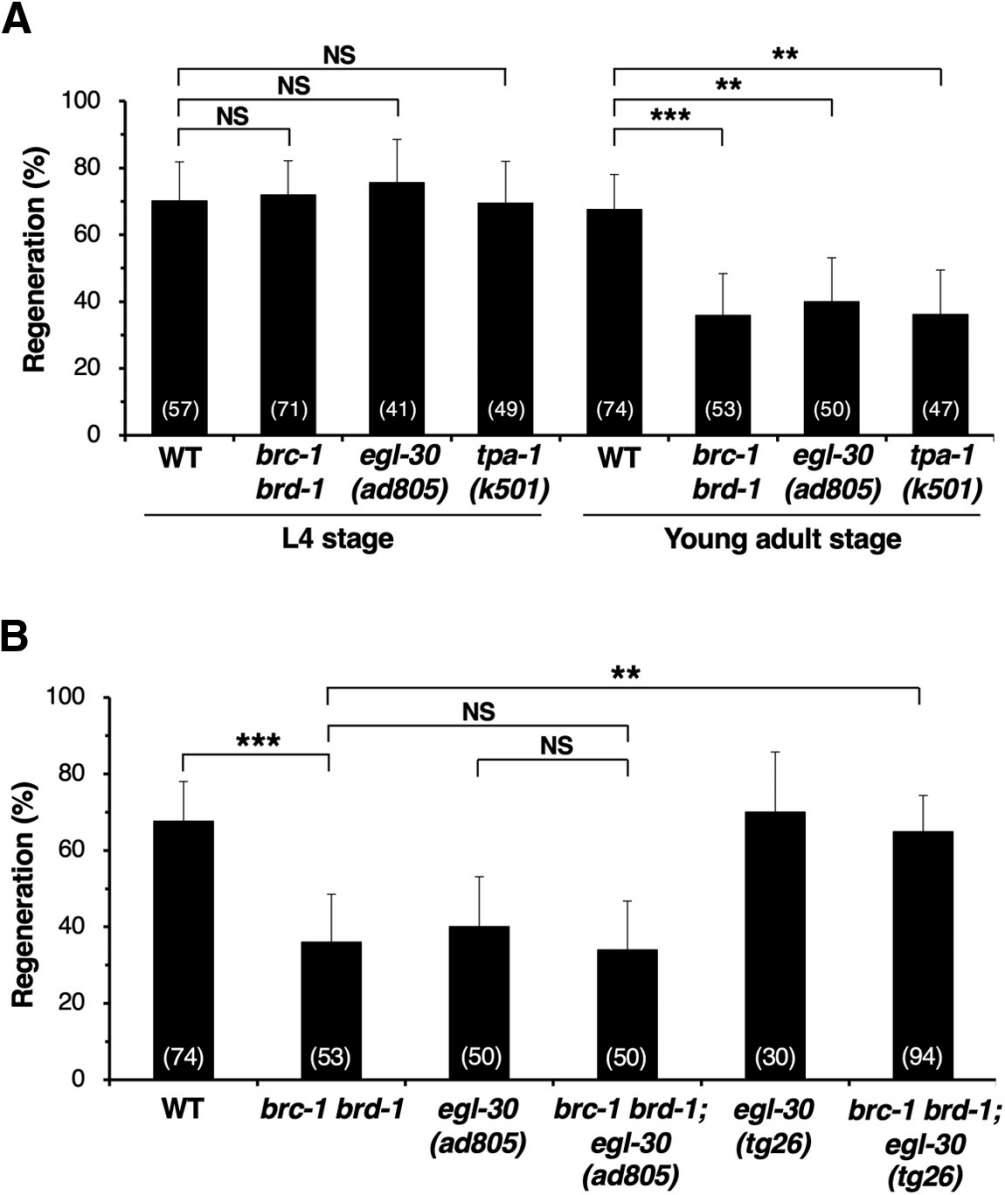
BRC-1–BRD-1 functions in the EGL-30 Gqα signaling pathway to regulate axon regeneration. ***A***, Percentages of axons that initiated regeneration 24 h after laser surgery at the L4 or young adult stage. The number of axons examined is shown. Error bars indicate 95% confidence intervals; **p < 0.01, ***p < 0.001, as determined by Fisher's exact test. NS, not significant. ***B***, Percentages of axons that initiated regeneration 24 h after laser surgery at the young adult stage. The number of axons examined is shown. Error bars indicate 95% confidence intervals; **p < 0.01, ***p < 0.001, as determined by Fisher's exact test. NS, not significant.

We have previously demonstrated that the CED-10 Rac type GTPase–MAX-2 and EGL-30 Gqα–TPA-1 PKC pathways regulate axon regeneration mainly at the L4 and young adult developmental stages, respectively (Pastuhov et al., 2012, 2016). It has been shown that *max-2* is expressed in ventral cord neurons during early development, but not at the young adult stage (Lucanic et al., 2006). This suggests that TPA-1 takes the place of MAX-2 to activate MLK-1 in axon regeneration at the adult stage. Therefore, we examined the relationship between life stage and axon regeneration in *brc-1(tm1145) brd-1(dw1)* double mutants. We found that axon regeneration in *brc-1(tm1145) brd-1(dw1)* mutants was reduced only in young adult animals and not in L4 larvae, a phenotype similar to that observed in *egl-30(ad805)* loss-of-function and *tpa-1(k501)* mutants (Fig. 4*A*; Table 2; Pastuhov et al., 2012, 2016). Thus, the BRC-1–BRD-1 complex participates in axon regeneration specifically at the adult stage.

This result raised the possibility that BRC-1–BRD-1 functions in the EGL-30 signaling pathway. To investigate this possibility, we examined the genetic interactions of *brc-1* and *brd-1* with *egl-30*. We found that the defect in axon regeneration caused by the *egl-30(ad805)* mutation was not enhanced by introduction of the *brc-1(tm1145) brd-1(dw1)* mutations (Fig. 4*B*; Table 2). This result supports the possibility that BRC-1–BRD-1 and EGL-30 act in the same pathway. Moreover, a gain-of-function *egl-30(tg26)* mutation was able to suppress the *brc-1 brd-1* phenotype (Fig. 4*B*; Table 2). These results suggest that BRC-1–BRD-1 promotes axon regeneration upstream of EGL-30. Alternatively, it is possible that BRC-1–BRD-1 enhances the EGL-30 pathway by inhibiting the action of a negative regulator of this signaling pathway.

### BRC-1–BRD-1 enhances the EGL-30 signaling pathway by downregulating DGK-3

**Figure 5.**
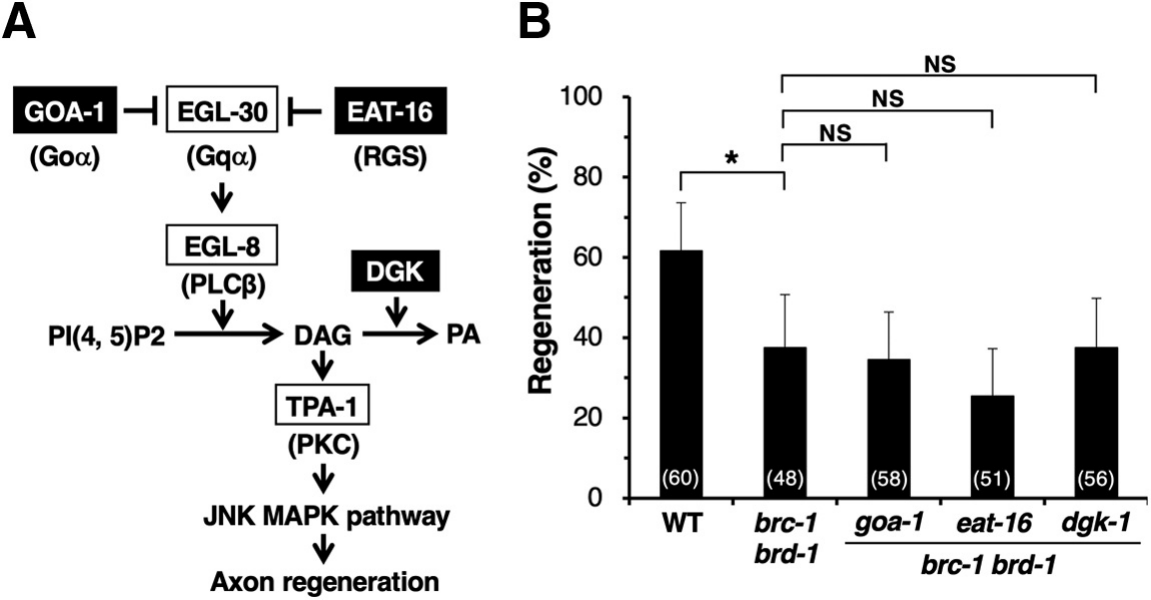
Effects of negative regulators in the EGL-30 pathway on BRC-1–BRD-1-mediated axon regeneration. ***A***, The EGL-30 pathway regulating axon regeneration. EGL-30 Gqα activates EGL-8 PLCβ, which in turn generates DAG from phosphatidylinositol bisphosphate [PI(4, 5)P2]. DAG activates TPA-1 PKC, resulting in activation of the JNK pathway to promote axon regeneration. GOA-1 Goα and EAT-16 RGS antagonize EGL-30 signaling. DGK downregulates the EGL-30 pathway by converting DAG to PA. ***B***, Percentages of axons that initiated regeneration 24 h after laser surgery at the young adult stage. The number of axons examined is shown. Error bars indicate 95% confidence intervals; *p < 0.05, as determined by Fisher's exact test. NS, not significant.

How does BRC-1–BRD-1 regulate the EGL-30 pathway in axon regeneration? The observation that BRC-1-associated E3-Ub ligase activity is required for axon regeneration (Fig. 2*B*) could suggest that some negative regulator of regeneration is inactivated by Ub-dependent protein degradation. It is known that GOA-1 Goα, the regulator of G-protein signaling (RGS) EAT-16, and DGK negatively regulate the EGL-30 pathway (Fig. 5*A*). We have previously demonstrated that the endocannabinoid anandamide inhibits axon regeneration via GOA-1, which antagonizes EGL-30 (Pastuhov et al., 2012). EAT-16 appears to negatively regulate EGL-30 by enhancing the rate of GTP hydrolysis (Chase et al., 2001). We examined whether BRC-1 promotes axon regeneration by downregulation of GOA-1 or EAT-16. However, we found that neither the *goa-1(n1134)* nor the *eat-16(nj8)* loss-of-function mutation suppressed the regeneration defect observed in *brc-1(tm1145) brd-1(dw1)* mutants (Fig. 5*B*; Table 2). Therefore, it is unlikely that GOA-1 or EAT-16 is a target for BRC-1–BRD-1-mediated degradation.

**Figure 6.**
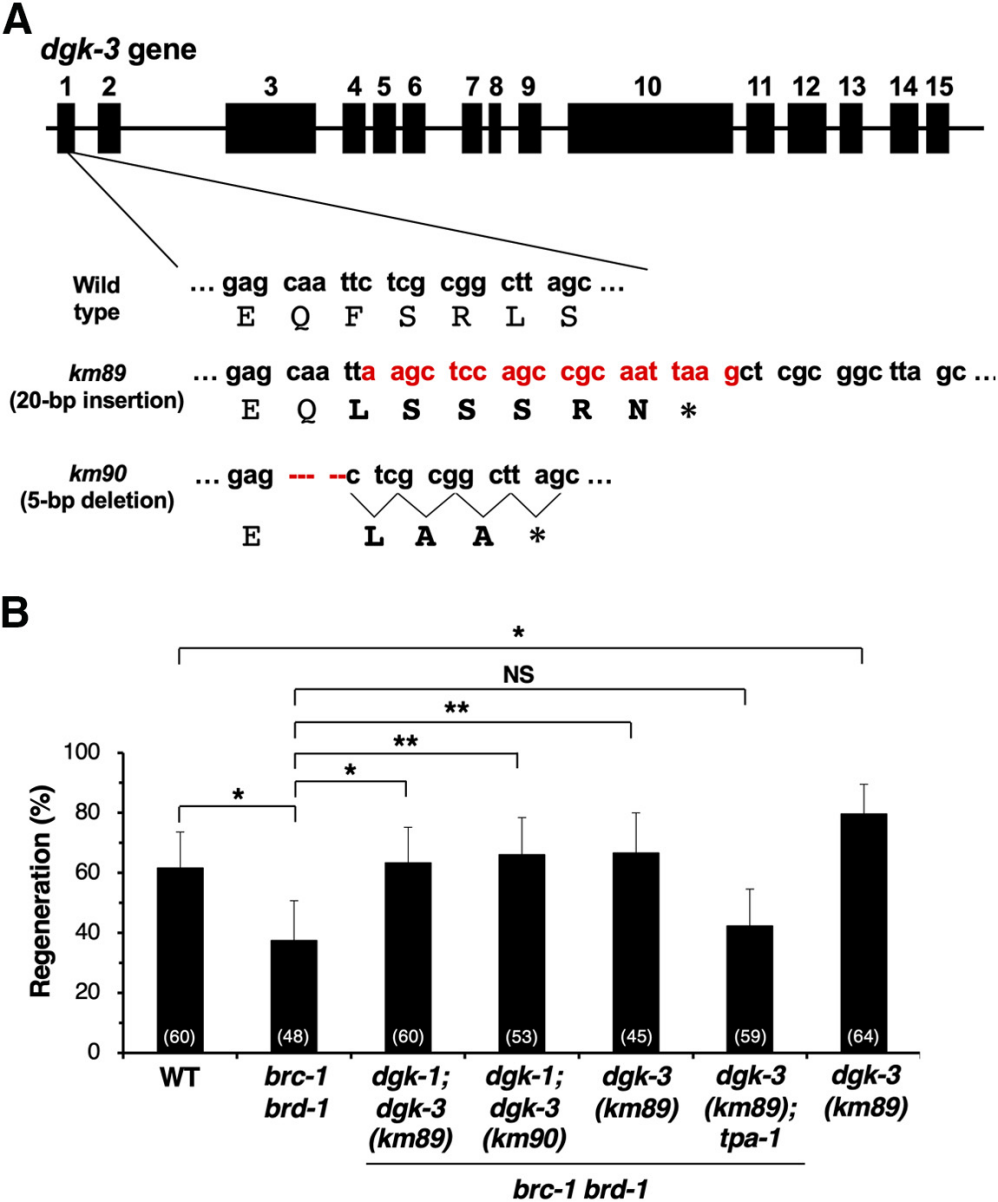
BRC-1–BRD-1 downregulates DGK-3 to promote axon regeneration. ***A***, Isolation of *dgk-3* mutants. Genomic structure of the *dgk-3* gene is shown. The *dgk-3(km89)* mutation is a 20-bp insertion (red nucleotides) that contains an in-frame premature stop codon (*), thus terminating translation in the middle of exon 1. The *dgk-3(km90)* mutation is a 5-bp deletion, causing a frameshift (bold amino acids) and premature stop codon (*) in exon 1. ***B***, Percentages of axons that initiated regeneration 24 h after laser surgery at the young adult stage. The number of axons examined is shown. Error bars indicate 95% confidence intervals; *p < 0.05, **p < 0.01, as determined by Fisher's exact test. NS, not significant.

DGK negatively regulates the EGL-30 pathway by converting DAG, an activator of TPA-1, into PA (Fig. 5*A*; Lackner et al., 1999). Indeed, we have previously reported that DGK-1, an ortholog of mammalian DGKθ, acts as a negative regulator of axon regeneration (Alam et al., 2016). However, we found that the *dgk-1(ok1462)* null mutation also failed to suppress the *brc-1(tm1145) brd-1(dw1)* phenotype of defective axon regeneration (Fig. 5*B*; Table 2). As the *C. elegans* genome contains five genes encoding DGKs, *dgk-1* to *dgk-5*, we considered the possibility that another DGK may be involved. Interestingly, Matsuki et al. (2006) recently reported that DGK-1 and DGK-3 function redundantly to reduce DAG levels and are required for olfactory adaptation. DGK-3 is an ortholog of mammalian DGKβ. To test whether *dgk-1; dgk-3* double mutations could suppress the *brc-1 brd-1* phenotype, we used CRISPR–Cas9 mutagenesis to generate two independent *dgk-3(km89)* and *dgk-3(km90)* null alleles (Fig. 6*A*) in the endogenous *dgk-3* locus of *brc-1(tm1145) brd-1(dw1)*; *dgk-1(ok1462)* mutants. We found that *dgk-3(km89)*; *dgk-1*(*ok1462*) and *dgk-3(km90)*; *dgk-1(ok1462)* mutations were able to suppress the *brc-1(tm1145) brd-1(dw1)* defect in axon regeneration (Fig. 6*B*; Table 2). To examine whether DGK-1 and DGK-3 redundantly regulate axon regeneration or whether DGK-3 does so alone, we constructed *brc-1(tm1145) brd-1(dw1) dgk-3(km89)* mutants. We found that the *dgk-3(km89)* single mutation was sufficient to suppress the *brc-1 brd-1* defect (Fig. 6*B*; Table 2). Axon regeneration was significantly improved with *dgk-3(km89)* single mutants compared with wild-type animals (Fig. 6*B*; Table 2). These results suggest that BRC-1–BRD-1 promotes axon regeneration by negatively regulating DGK-3, thereby ensuring elevated DAG levels, which activates TPA-1. Consistent with this, the *dgk-3(km89)* mutation failed to suppress the defect in axon regeneration in *brc-1 brd-1; tpa-1* mutants (Fig. 6*B*; Table 2).

### BRC-1–BRD-1 poly-ubiquitylates DGK-3, leading to its degradation

**Figure 7.**
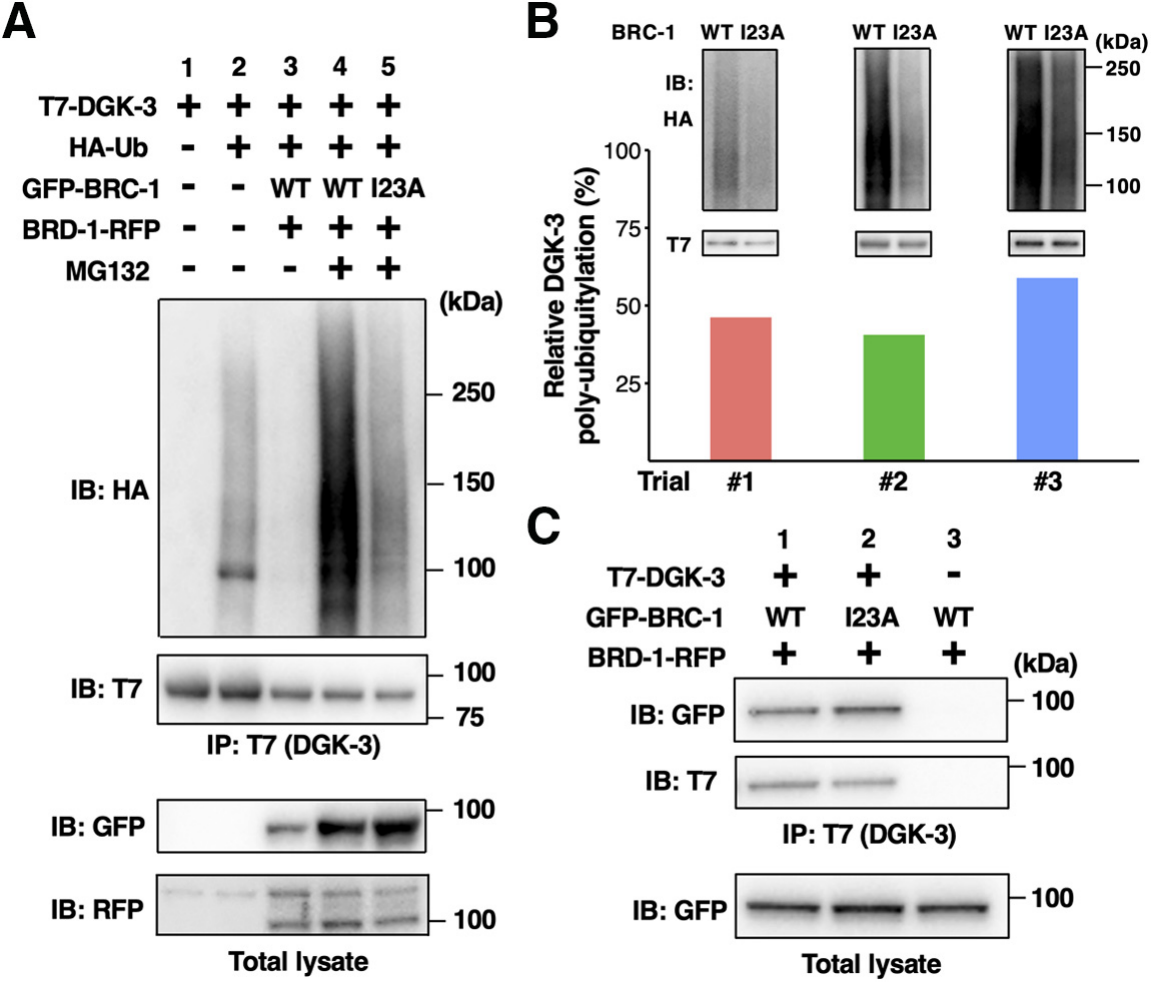
BRC-1–BRD-1 mediates poly-ubiquitylation of DGK-3. ***A***, Poly-ubiquitylation of DGK-3 by BRC-1–BRD-1. COS-7 cells were transfected with T7-DGK-3, HA-Ub, GFP-BRC-1, and BRD-1-RFP, as indicated. Cells were incubated with or without MG132. Cell lysates were immunoprecipitated (IP) with anti-T7 antibody and immunoblotted (IB) with anti-HA and anti-T7 antibodies. Total lysates were analyzed by IB with anti-GFP and anti-RFP antibodies. The experiment was done in triplicate with similar results, shown here for trial #2. ***B***, Comparison of DGK-3 poly-ubiquitylation levels. The DGK-3 poly-ubiquitylation experiment was performed three times and each bar represents the result of each trial (#1–#3). Data represent the percentage of normalized poly-ubiquitylated DGK-3 in lane 5 relative to that found in lane 4. The blots in lanes 4 and 5 of ***A*** from three trials are shown in the upper part. ***C***, Interaction of DGK-3 with BRC-1. COS-7 cells were co-transfected with T7-DGK-3, GFP-BRC-1, and BRD-1-RFP, as indicated. Cells were then incubated with MG132. Cell lysates were immunoprecipitated (IP) with anti-T7 antibody and immunoblotted (IB) with anti-GFP and anti-T7 antibodies. Total lysates were analyzed by IB with anti-GFP antibody.

The genetic analysis described above raised the possibility that BRC-1–BRD-1 could act as an E3-Ub ligase to mediate ubiquitylation of DGK-3, thus promoting its degradation. To test this hypothesis, we examined whether BRC-1–BRD-1 ubiquitylates DGK-3 in mammalian cell cultures. We co-expressed T7-tagged DGK-3 and HA-tagged Ub in COS-7 cells, immunoprecipitated cell lysates with anti-T7 antibody and immunoblotted with anti-HA antibody. We detected mono-ubiquitylation and weak poly-ubiquitylation of DGK-3 (Fig. 7*A*, lanes 1 and 2), suggesting that there is some endogenous E3-Ub ligase in COS-7 cells that can ubiquitylate DGK-3. We next evaluated whether BRC-1–BRD-1 could stimulate the ubiquitylation of DGK-3. T7-DGK-3 and HA-Ub were co-transfected with GFP-BRC-1 and BRD-1-RFP into COS-7 cells. We found that co-expression of BRC-1 and BRD-1 decreased the levels of poly-ubiquitylated DGK-3 (Fig. 7*A*, lane 3), suggesting that BRC-1–BRD-1 promotes the degradation of ubiquitylated DGK-3. Consistent with this possibility, when cells were treated with MG132, a specific inhibitor of the 26S proteasome, the level of poly-ubiquitylated DGK-3 clearly increased (Fig. 7*A*, lane 4). However, instead of wild-type BRC-1, co-expressing BRD-1 with mutant BRC-1(I23A), which is defective in E3-Ub ligase activity, resulted in decreased levels of poly-ubiquitylated DGK-3 in the presence of MG132 (Fig. 7*A*,*B*). These results suggest that BRC-1–BRD-1 controls DGK-3 protein levels through proteasome-mediated degradation.

To determine whether DGK-3 interacts with BRC-1, COS-7 cells were transiently transfected with T7-DGK-3, GFP-BRC-1, and BRD-1-RFP, and then treated with MG132 to inhibit DGK-3 degradation. Co-immunoprecipitation experiments revealed an interaction between DGK-3 and BRC-1 (Fig. 7*C*, lane 1). Similar results were observed between DGK-3 and BRC-1(I23A) (Fig. 7*C*, lane 2). Therefore, the E3-Ub ligase activity of BRC-1 is not required to interact with DGK-3. These results indicate that BRC-1 interacts with and poly-ubiquitylates DGK-3 for degradation.

**Figure 8.**
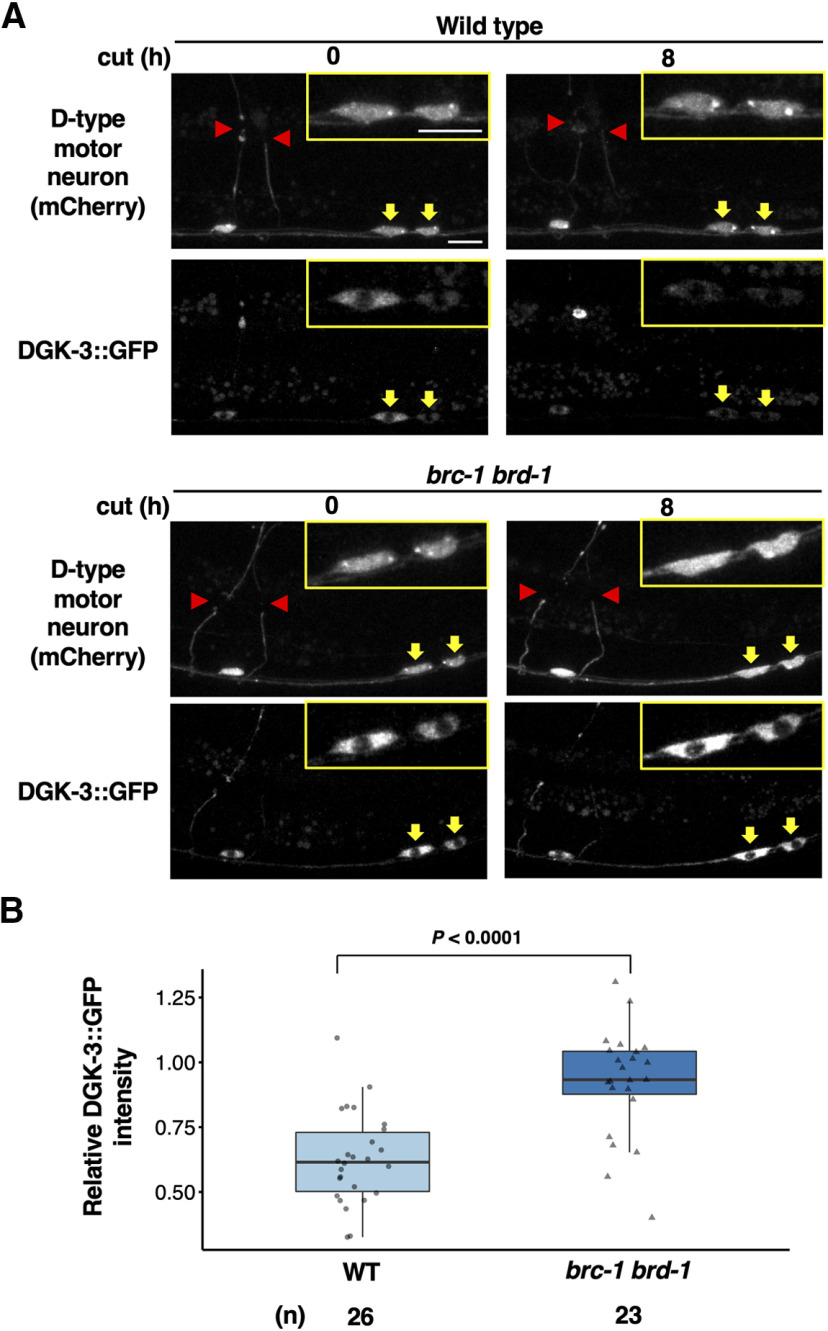
BRC-1–BRD-1 promotes axotomy induced degradation of cytoplasmic DGK-3. ***A***, Fluorescent images of wild-type and *brc-1 brd-1* mutant animals expressing *Punc-47::mcherry* (D-type motor neuron, top) and *Punc-25::dgk-3::GFP* (bottom) are shown. Images were taken at 0 or 8 h after laser surgery. Red arrowheads indicate the tip of the severed axons. Yellow arrows indicate cell bodies corresponding to the severed axons and their magnification is shown in the insets. Scale bar: 10 µm. ***B***, Quantification of DGK-3::GFP fluorescence levels in cytoplasm of D-type neurons. Relative DGK-3 intensity was calculated as a fraction of the relative DGK-3::GFP intensity 8 h after laser surgery divided by the corresponding value at 0 h postaxotomy. Data are presented as a box-plot representing median (thick line within the box) and interquartile range (edge of box) with individual data points. The number (*n*) of axons examined is shown. Statistical significance was determined by Wilcoxon rank-sum test.

Next, we investigated whether BRC-1–BRD-1 regulates DGK-3 levels in animals by expressing GFP-fused DGK-3 in D-type motor neurons using the *unc-25* promoter. In wild-type animals, DGK-3::GFP was uniformly distributed in the cytoplasm of D-type neuron cell bodies (Fig. 8*A*). Following axon laser ablation, fluorescence intensity of DGK-3::GFP in the cytoplasm of D-type neurons was significantly decreased (Fig. 8*A*,*B*). In contrast, we found that the *brc-1(tm1145) brd-1(dw1)* mutations resulted in significant stabilization of cytosolic DGK-3::GFP levels (Fig. 8*A*,*B*). Thus, BRC-1–BRD-1 is involved in axon injury-induced destabilization of DGK-3 in animals. These results suggest that increases in DGK-3 protein levels in *brc-1 brd-1* mutants lead to a decrease in DAG levels, which eventually results in the inhibition of the TPA-1 PKC signaling pathway.

### PKA phosphorylation induces cytoplasmic localization of BRC-1

**Figure 9.**
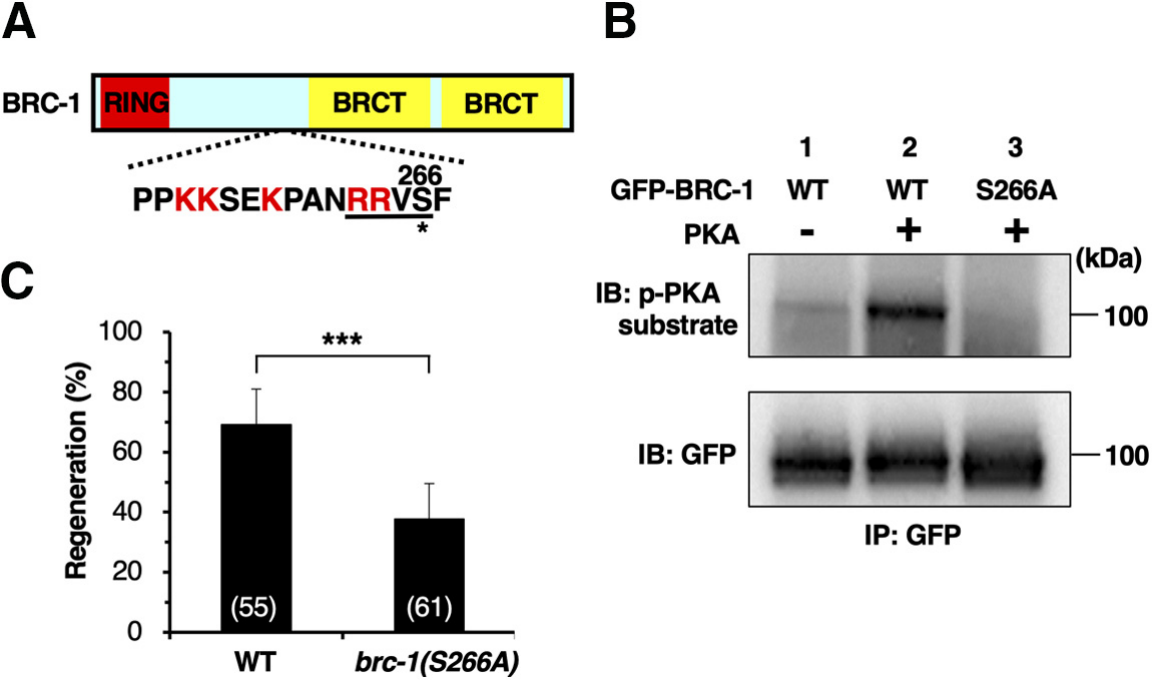
PKA phosphorylates BRC-1. ***A***, A schematic diagram of BRC-1. RING finger domain is shown in red, and BRCT domains in yellow. The amino acid sequences around a PKA phosphorylation consensus site (underline) and a putative nuclear localization signal (red characters) are shown below. The Ser-266 residue is indicated by an asterisk. ***B***, PKA phosphorylates BRC-1 at Ser-266 *in vitro*. *In vitro* phosphorylation of BRC-1 by PKA is shown. COS-7 cells were transfected with GFP-BRC-1 (WT) or GFP-BRC-1(S266A), and cell lysates were immunoprecipitated (IP) with anti-GFP antibody. The immunoprecipitates were subjected to *in vitro* kinase assay using active recombinant PKA. Phosphorylated BRC-1 was detected by immunoblotting (IB) with anti-phospho-PKA substrate rabbit monoclonal antibody. ***C***, Percentages of axons that initiated regeneration 24 h after laser surgery at the young adult stage. The number of axons examined is shown. Error bar indicates 95% confidence interval; ***p < 0.001, as determined by Fisher's exact test.

How is BRC-1 function regulated in axon regeneration? Upon axon severance, intracellular levels of cAMP increase and PKA is activated (Neumann et al., 2002; Bhatt et al., 2004). Interestingly, BRC-1 contains a PKA phosphorylation consensus motif (Arg-Arg-Xxx-Ser) at Ser-266 (Fig. 9*A*). We therefore asked whether PKA phosphorylates BRC-1 at this residue. We performed *in vitro* kinase assays with active PKA and immuno-purified GFP-BRC-1 from COS-7 cells. Western blot analysis using an antibody recognizing phosphorylated PKA substrates revealed PKA phosphorylation of GFP-BRC-1 (Fig. 9*B*, lanes 1 and 2). To determine whether PKA can phosphorylate BRC-1 at Ser-266, we generated a mutant form of BRC-1 [BRC-1(S266A)], in which Ser-266 was replaced with alanine. *In vitro* kinase assays revealed that the S266A mutation abolished the phosphorylation of BRC-1 by PKA (Fig. 9*B*, lane 3). These results demonstrate that PKA phosphorylates Ser-266 of BRC-1 *in vitro*. In order to address the physiological significance of this phosphorylation, we used the CRISPR–Cas9 system to engineer a non-phosphorylatable *brc-1(S266A)* mutant, replacing the codon encoding the Ser-266 residue with an alanine codon in the endogenous *brc-1* locus. We found that axon regeneration was significantly reduced in *brc-1(S266A)* mutants (Fig. 9*C*; Table 2). This result indicates that Ser-266 phosphorylation is important for activation of the regeneration pathway by BRC-1.

**Figure 10.**
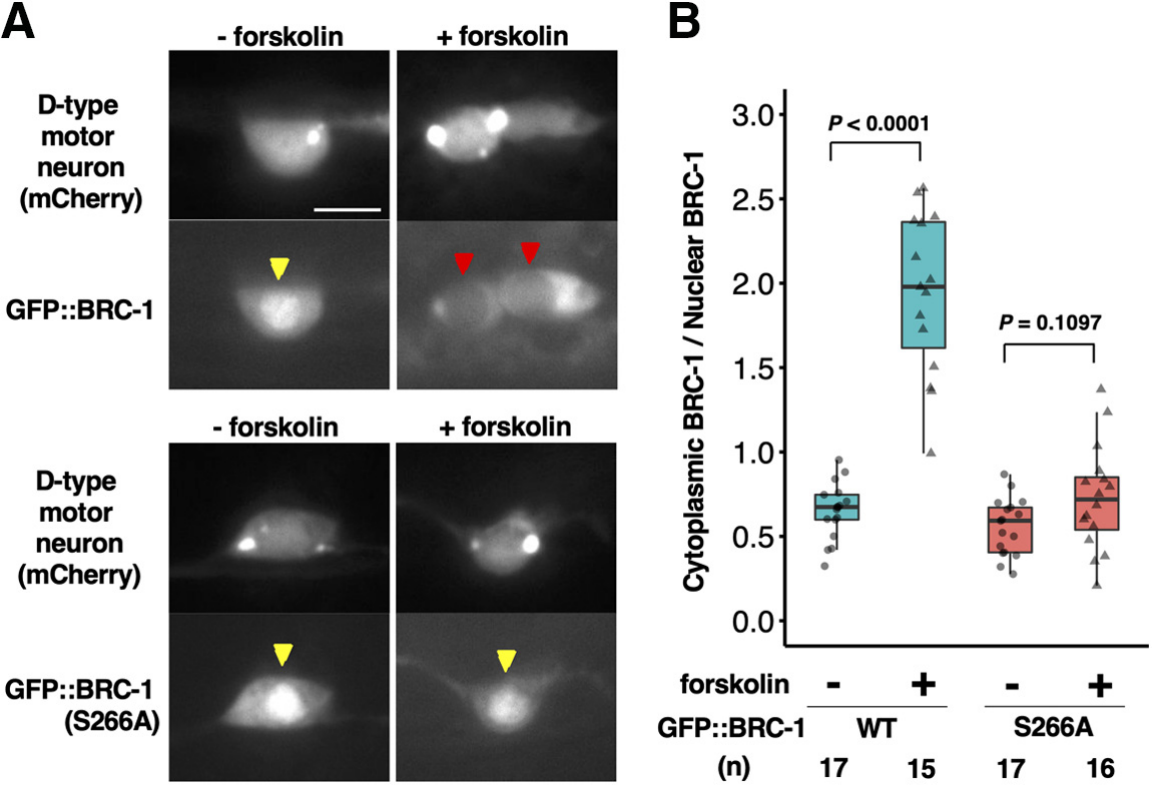
PKA phosphorylation induces cytoplasmic localization of BRC-1. ***A***, Localization of BRC-1 in response to PKA activation. Fluorescent images of wild-type animals expressing *Punc-47::mcherry* (D-type motor neuron, top) and *Punc-25::GFP::brc-1* or *Punc-25::GFP::brc-1(S266A)* (bottom) with or without forskolin treatment are shown. Red and yellow arrowheads indicate cell nucleus. Scale bar: 10 µm. ***B***, Quantification of GFP::BRC-1 fluorescence levels in D-type neurons with or without forskolin treatment. The cytoplasmic-to-nuclear ratio of GFP::BRC-1 signal was calculated as a fraction of the relative GFP::BRC-1 intensity in the cytoplasm divided by the corresponding value in the nucleus. Data are presented as a box-plot representing median (thick line within the box) and interquartile range (edge of box) with individual data points. The number (*n*) of cell bodies examined is shown. Statistical significance was determined by Wilcoxon rank-sum test.

We next examined how PKA-mediated phosphorylation might regulate BRC-1 in axon regeneration. Interestingly, the PKA phosphorylation site of BRC-1 is in a putative nuclear localization signal (NLS) sequence (Fig. 9*A*), raising the possibility that phosphorylation of BRC-1 might impact its localization. We investigated this possibility by monitoring GFP::BRC-1 localization during activation of PKA. Under normal conditions, GFP::BRC-1 was predominantly localized in the nucleus (Fig. 10*A*,*B*). Treatment of animals with forskolin is expected to cause an increase in cAMP levels by activating adenylyl cyclase and concomitantly PKA (Ghosh-Roy et al., 2010). We found that forskolin treatment strongly induced cytoplasmic localization of GFP::BRC-1 (Fig. 10*A*,*B*). In contrast, forskolin was unable to induce cytoplasmic accumulation of the GFP::BRC-1(S266A) mutant (Fig. 10*A*,*B*), suggesting that PKA phosphorylation of the Ser-266 site is required for the translocation of BRC-1 from the nucleus to the cytoplasm. Thus, by altering its subcellular localization, the phosphorylation of BRC-1 at Ser-266 can regulate axon regeneration.

## Discussion

*BRCA1* and *BRCA2* genes were identified as causative genes for early-onset hereditary breast cancer (Fackenthal and Olopade, 2007). *BRCA*-deficient cells use error-prone DNA-repair pathways, which cause increased genomic instability (Scully and Livingston, 2000; Moynahan and Jasin, 2010; Ceccaldi et al., 2016). However, recent studies have identified new functions of BRCA1 and BRCA2 in the regulation of transcription and RNA processing relevant to their tumor-suppressive activity (Kleiman et al., 2005). Previous studies have established that the *C. elegans* orthologs, BRC-1 (for BRCA1) and BRC-2 (for BRCA2), possess many functional similarities with their human counterparts, including DNA damage repair, homologous recombination, and meiosis (Martin et al., 2005; Polanowska et al., 2006; Adamo et al., 2008; Janisiw et al., 2018; Li et al., 2018). Therefore, *C. elegans* has proven to be a very useful model system for studying the function and signaling pathways of BRCA1 and BRCA2.

**Figure 11.**
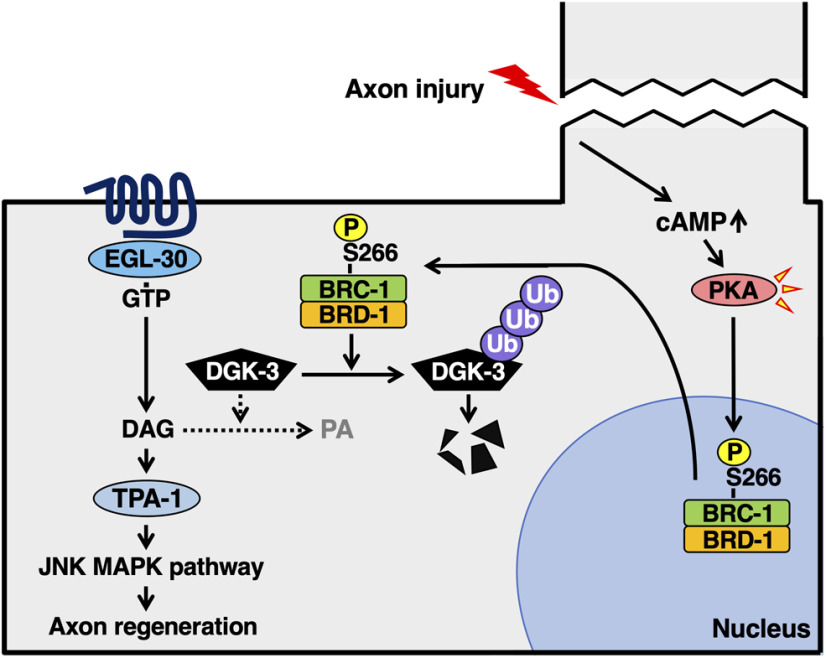
Schematic model for the regulation of axon regeneration by BRC-1–BRD-1. Under normal conditions, BRC-1–BRD-1 is localized in the nucleus. In response to axon injury, PKA phosphorylation of BRC-1 Ser-266 induces the translocalization of BRC-1–BRD-1 to the cytoplasm. The BRC-1–BRD-1 complex poly-ubiquitylates DGK-3, resulting in its degradation. BRC-1–BRD-1 enhances the EGL-30 signaling pathway to promote axon regeneration.

We have recently found that BRC-2 regulates axon regeneration of postdifferentiated GABAergic D-type motor neurons after injury through the Rho GTPase signaling pathway (Shimizu et al., 2018). In the present study, we find that BRC-1 is also involved in axon regeneration. In humans, BRCA1 exists mostly in a heterodimeric complex with its binding partner BARD1 (Wu et al., 1996). Similarly, BRC-1 forms a complex with the *C. elegans* BARD1 ortholog BRD-1, and BRD-1 is also required for the regeneration of severed axons. However, the site of action of BRC-1–BRD-1 in the regulation of axon regeneration is different from that of BRC-2. BRC-1–BRD-1 participates in adult-specific axon regeneration regulated by the EGL-30 Gqα signaling pathway. Activated EGL-30 signaling induces increased production of DAG, which in turn activates TPA-1 PKC (Lackner et al., 1999). TPA-1 phosphorylates and activates MLK-1 MAPKKK to promote axon regeneration (Pastuhov et al., 2012). DGK converts DAG to PA (Miller et al., 1999); thus, inactivation of DGK activity results in elevated DAG levels. The BRC-1–BRD-1 complex enhances the EGL-30 pathway by poly-ubiquitylating DGK-3, which results in its degradation through the 26S proteasome pathway (Fig. 11). Based on this possibility, the recovery of axon regeneration in *brc-1 brd-1* mutants by gain-of-function *egl-30* or *dgk-3* deletion mutations could be a compensatory effect. The *brc-1 brd-1* mutant is defective in DGK-3 degradation, resulting in reduced DAG levels. The *egl-30* or *dgk-3* mutation can suppress the *brc-1 brd-1* deficiency by increasing DAG levels.

In mammals, at least 10 DGK isoforms have been reported, and their expression patterns or interactors differ among isoforms (Topham and Epand, 2009; Ishisaka and Hara, 2014). *C. elegans* contains five *dgk* genes (*dgk-1* to *dgk-5*), each encoding a different isoform corresponding to mammalian DGK. To date, DGK-1 and DGK-3 have been shown to play roles in the nervous system. The *dgk-1* mutation enhances DAG signaling in several sensory and motor neurons (Miller et al., 1999; Matsuki et al., 2006), whereas DGK-3 functions in AFD sensory neurons and modulates thermotactic behavior (Biron et al., 2006). In AWC chemosensory neurons, DGK-1 and DGK-3 function redundantly to control olfactory adaptation (Matsuki et al., 2006). In this study, we observed that deletion of *dgk-3* alone is sufficient to reverse the regeneration defect of *brc-1 brd-1* mutants. In contrast, the *dgk-1* single knockout in *brc-1 brd-1* mutants has no effect. Therefore, BRC-1–BRD-1 specifically inhibits DGK-3; however, disruption of *dgk-1* may cause an increase in DAG levels, resulting in suppression of the *brc-1 brd-1* phenotype. Recently, we demonstrated that the *C. elegans* small GTPase RHO-1 promotes axon regeneration by inactivating DGK-1, leading to DAG upregulation in D-type motor neurons (Alam et al., 2016). Thus, these results suggest that deletion of *dgk-1* does not further increase DAG level in D-type neurons of *brc-1 brd-1* mutants because DGK-1 activity is already inhibited by RHO-1 during axon regeneration.

Since tumor-derived BRCA1 mutations eliminate E3-Ub ligase activity (Baer and Ludwig, 2002), it is clear that this activity of mammalian BRCA1–BARD1 is of critical functional importance. The identification of targets of BRCA1–BARD1-dependent ubiquitylation would inform our understanding of the role of BRCA1–BARD1 in tumorigenesis, however at present these targets are unknown. We show here that the E3-Ub ligase activity of BRC-1–BRD-1 is critical for its function in axon regeneration. We identify DGK-3 as a specific target for BRC-1–BRD-1-mediated ubiquitylation, which may suggest that mammalian BRCA1–BARD1 function in diverse cellular processes could involve ubiquitylation of DGKs. Indeed, the mammalian DGKζ, whose function is linked to cancer cell growth and survival, is poly-ubiquitylated and degraded through the proteasome system (Okada et al., 2012; Torres-Ayuso et al., 2015). It would be interesting to ask whether ubiquitylation of DGKζ is mediated by the BRCA1–BARD1 complex. Recently, Krishnan et al. (2018) reported that mammalian BRCA1 is also involved in axon regeneration of adult peripheral neurons. Axon injury triggers BRCA1-dependent DNA damage response signaling in the neuronal soma. In contrast to BRC-1 in *C. elegans*, BRCA1 is mainly localized in the cytoplasm, and axotomy induces translocation to the nucleus. As a result, BRCA1 supports the transcriptional program of injured neurons. Thus, the targets of BRCA1/BRC-1 in the regulation of axon regeneration may be different between mammals and *C. elegans*.

BRCA1–BARD1-dependent ubiquitylation events are regulated at sites of DNA damage. Human BRCA1 is directly phosphorylated by ATM and ATR kinases in response to DNA damage (Cortez et al., 1999), suggesting that this phosphorylation regulates the retention of BRCA1–BARD1 at sites of DNA damage. It is therefore plausible that axon injury regulates BRC-1–BRD-1-dependent ubiquitylation of DGK-3 through phosphorylation. Indeed, we show that PKA activated by axon injury phosphorylates BRC-1 at Ser-266 and this induces the translocation of BRC-1 from the nucleus to the cytoplasm. The Ser-266 site is located in a putative NLS sequence of BRC-1. Consistent with this, the BRC-1(S266A) mutant remains localized to the nucleus even with PKA activation. Our results suggest the following model for the control of BRC-1 localization (Fig. 11). Under normal conditions, BRC-1 is mostly localized in the nucleus. In response to axon injury, PKA phosphorylation induces the translocalization of BRC-1 to the cytoplasm. Because DGK-3 is present in the cytoplasm, we postulate that phosphorylation-dependent cytoplasmic accumulation of BRC-1 results in enhanced poly-ubiquitylation of DGK-3.
